# Effect of Long Noncoding RNA HULC on Proliferation, Migration, and Invasion of Osteosarcoma Cells

**DOI:** 10.1155/2022/7526731

**Published:** 2022-09-30

**Authors:** Ran Zhao, Xin Zhou, Wencan Zhang, Le Li

**Affiliations:** ^1^Department of Burns and Plastic Surgery, Shandong Provincial Hospital Affiliated to Shandong First Medical University, Jinan, 250021 Shandong, China; ^2^Department of Orthopedic Surgery, Qilu Hospital of Shandong University, Jinan, 250021 Shandong, China

## Abstract

**Background:**

Previous studies had shown that lncRNA HULC exhibited different effects in human cancers. However, the role of HULC was not reported in osteosarcoma. Hence, we designed this research to explore the function of HULC in osteosarcoma.

**Methods:**

Firstly, HULC expression was measured in osteosarcoma tissues and cells via the RT-qPCR assay. The protein expression was detected through western blot. Then, CCK-8 and Transwell assays were conducted to measure cell proliferation, migration, and invasion.

**Results:**

The expression of HULC was obviously higher in osteosarcoma tissues and cells compared with normal control. Moreover, cell proliferation, migration, and invasion were inhibited by HULC knockdown in osteosarcoma cells. HULC overexpression markedly increased osteosarcoma cell proliferation and tumor size in vivo. Furthermore, HULC increased the activity of AKT-PI3K-mTOR pathway by blocking PTEN in osteosarcoma cells. LY294002 inhibited the phosphorylation of AKT, mTOR, and PI3K. Overexpressing HULC enhanced cell migration and invasion of SAOS-2 cells and MG63 cells, while LY294002 reversed the effects.

**Conclusion:**

HULC enhanced the progression of osteosarcoma through targeting PTEN.

## 1. Introduction

Osteosarcoma is caused by bone cell abnormal differentiation and proliferation. Consistent with medicine information, the incidence of osteogenic sarcoma is around 0.2–3/100000 per annum [1]. It is the foremost common primary bone tumor in youngsters and adolescents with a high degree of malignancy. Osteosarcoma typically shows a high tendency to pathologic process spread [2]. Osteosarcoma is commonly treated with a mixture of therapies that may embody surgery, therapy, and radiotherapy [3]. However, in recent years, the survival rate of patients with osteosarcoma amid distant metastasis has not been considerably improved, and also the impact of therapy has not been considerably improved, and also the treatment of osteosarcoma remains controversial [4, 5]. Thus, new therapeutic targets ought to be found to produce clinical treatment choices to enhance the survival rate [6].

High-throughput transcriptome sequencing analysis has shown that only 2% of the human genome can be transcripted into proteins, and 98% can be transcripted into noncoding RNAs [7]. The function of these noncoding RNAs is still poorly understand. The long noncoding RNA is a type of noncoding RNA that is longer than 200 nt [8]. lncRNAs have been shown to be involved in a large number of cellular processes, such as cell proliferation, transcriptional and posttranscriptional modification, epigenetic modification, and invasion [9]. The large number of studies shows that lncRNA could play an important role in many types of cancer [10]. For example, Chen et al. showed that lncRNA HULC was overexpressed in epithelial ovarian carcinoma and target ATG7 [11]. Zheng et al. showed that lncRNA HULC was highly expressed in HeLa cells and promotes cell migration and invasion [12].

lncRNA HULC was first reported by Panzitt et al. in 2007, which was overexpressed lncRNA in human hepatocellular carcinoma [13]. HULC gene is located on chromosome 6p24.3 with approximately 500 nucleotides in length and contains two exons [14]. Since then, the function of HULC has been demonstrated in multiple cancer types [15, 16]. HULC could promote cancer cell survival, proliferation, and invasion. These studies indicated that HULC played an important role in the development of cancer [16, 17]. HULC was overexpressed in human osteosarcoma cell which has been shown in the previous studies. However, the mechanism of HULC involved in the progression of osteosarcoma is unknown, and the interaction of HULC and PTEN has not been proved in osteosarcoma.

Therefore, we investigated the interaction of HULC and PTEN in promoting osteosarcoma cell proliferation and migration.

## 2. Materials and Methods

### 2.1. Cell Lines and Culture

The human osteosarcoma cell lines, including Saos-2, U-2OS, and MG63, were selected as subjects for subsequent analysis. All cells were purchased from the American Type Culture Collection (ATCC) (Manassas, VA, USA).

### 2.2. RNA Extraction and Quantitative Real-Time PCR (qRT-PCR)

Total RNA was extracted by TRIzol reagent (Invitrogen Inc., Carlsbad, CA, USA). Subsequently, the RNA was reversely transcribed to the cDNA. The M-MLV reverse transcriptase (Promega, Madison, WI, USA) was used for reverse transcription reaction. SYBR-Green Real-Time Master Mix (Toyobo, Tokyo, Japan) was applied for qRT-PCR reaction. Finally, the data were calculated with 2^−*ΔΔ*Ct^ method.

HULC P1: 5′-AACCTCCAGAACTGTGAT-3′ and HULC P2: 5′-CATAATTCAGGGAGAAAG-3′

*β*-Actin P1: 5′-CTTCCTTCCTGGGCATGGAG-3′ and *β*-actin P2: 5′-GGAACGCTTCACGAATTTGC-3′

### 2.3. Western Blot

The cells were treated with RAPA buffer (Beyotime, Jiangsu, China) on ice for 30 min. BCA kit (Pierce, Rockford, IL, USA) was applied for the measurement of protein concentration. Subsequently, total proteins were separated by 12% SDS-polyacrylamide gel electrophoresis (SDS-PAGE) and then were transferred on polyvinylidene fluoride membranes (Millipore, Billerica, MA, USA). The membranes were treated with 5% fat-free milk for 2 h and then were incubated with the antibodies of AKT (PTG, USA, 1 : 1000), p-AKT (PTG, USA, 1 : 10000), PI3K (CST, USA, 1 : 1000), p-PI3K (CST, USA, 1 : 1000), mTOR (PTG, USA, 1 : 20000), p-mTOR (PTG, USA, 1 : 10000), PTEN (ABclonal, China, 1 : 1000), or *β*-actin (CST, USA, 1 : 1000). After washing and incubating with the second antibodies, the abundance of the proteins was analyzed by enhanced chemiluminescence (ECL).

### 2.4. shRNA Transfection

In short, the specific oligonucleotides targeting HULC were synthesized by Genepharmacy Technology (China): sense: 5′-GATCCGCCACATGAACGCCCAGAGATTTTCAAGAGAAATCTCTGGGCGTTCATGTGGTTTTTTG-3′ and antisense: 5′-AATTCAAAAAACCACATGAACGCCCAGAGATTTCTCTTGAAAATCTCTGGGCGTTCATGTGGCG-3′. After that, the plasmids and control were transfected into Saos-2 and MG63 cells. RT-qPCR was performed to confirm the knockdown efficiency of HULC.

### 2.5. Cell Viability Assay

The cell viability was detected using Cell Counting Kit-8 (CCK-8). Briefly, 1 × 10^4^ cells were seeded into 96-well plates and cultured for 1, 2, 3, and 4 d. Subsequently, osteosarcoma cells were incubated with CCK-8 at 37°C for 1 h. The absorbency at 450 nm of the cells was measured with microplate reader Thermo Plate (Rayto Life and Analytical Sciences Co., Ltd., Germany).

### 2.6. Cell Migration and Invasion Assays

For migration assay, 2 × 10^4^ cells and 100 *μ*l serum-free medium were injected into upper chamber. For Transwell invasion assay, Matrigel-coated chamber was used for invasion assay, and 3 × 10^4^ cells were plated in 100 *μ*l serum-free medium in the upper. In both assays, 500 *μ*l of the medium and 20% FBS were added to the lower chamber. Subsequently, the cells were cultured at 37°C and 5% CO_2_ for 24 h. After that, the cells were fixed with 100% methanol for 30 min, and then, the cells in upper chamber were removed. Finally, the cells were stained with 0.5% crystal violet (Sigma, St. Louis, MO, USA) for 20 min for cell count.

### 2.7. Xenograft Tumor Formation

Male BALB/c nude mice (6 weeks) were obtained from HFK Biosciences and maintained under pathogen-free conditions. The experiment was approved by the Shandong Provincial Hospital Affiliated to Shandong First Medical University. For tumor propagation analysis, the mice accepted the subcutaneous injection including 2 × 10^6^ HULC overexpression cells. After 5 week, the weight of the tumors was measured.

### 2.8. Statistical Analysis

SPSS 20.0 (SPSS Inc., Chicago, IL, USA) was applied for data analyses. The Kaplan-Meier method and log-rank test were applied for the visualization of survival curves. Moreover, one-way analyses of variance and two-tailed Student's *t*-tests were applied for difference analysis. *P* < 0.05 was considered statistically significant.

## 3. Results

### 3.1. lncRNA HULC Was Increased in Osteosarcoma Tissue

**Figure 1:**
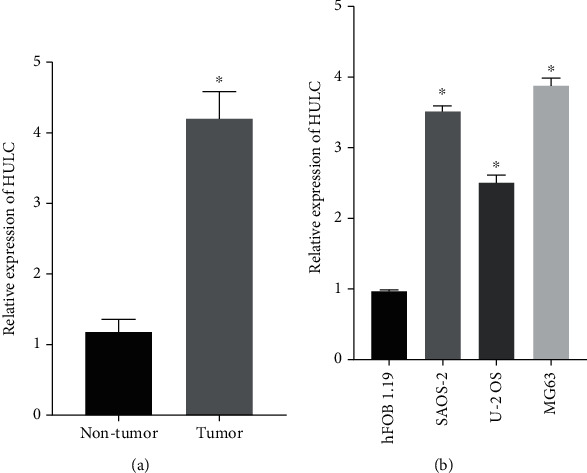
lncRNA HULC expression was increased in osteosarcoma tissue and cells. The expressions of HULC in osteosarcoma tissues (a) and cell lines (b) were detected by RT-qPCR. ^∗^*P* < 0.05.

First, the expression of lncRNA HULC abundance was examined in osteosarcoma tissue and normal tissue using RT-qPCR. The expression of HULC in tumor tissues was higher than normal tissues (Figure 1(a)). Similarly, the expression of HULC was also increased in tumor cell lines Saos-2, MG63, and U-2OS in contrast to normal cell (hFOB 1.19) (Figure 1(c)).

### 3.2. HULC Knockdown Inhibited the Malignant Behaviors of Osteosarcoma Cells

**Figure 2:**
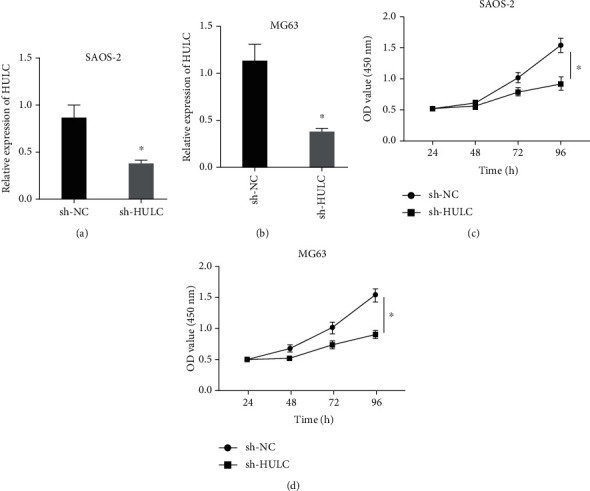
Downregulation of HULC impeded the proliferation of osteosarcoma cells. (a, b) The relative expression of HULC in cells was detected in SAOS-2 (a) and MG63 cells (b) by RT-qPCR. (c, d) Cell viability in SAOS-2 (c) and MG63 cells (d) was measured by CCK-8 assay. ^∗^*P* < 0.05.

**Figure 3:**
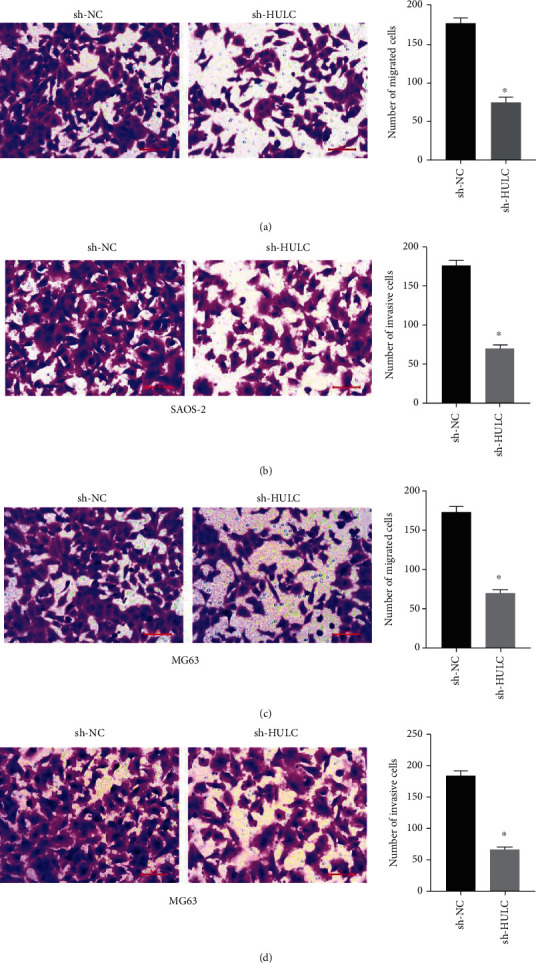
Decreased HULC impeded the malignant behaviors of osteosarcoma cells. (a, b) Cell migration (a) and invasion (b) assays in SAOS-2 cells were detected by Transwell assay. (c, d) Cell migration (c) and invasion (d) assays in MG63 cells were measured by Transwell assay. ^∗^*P* < 0.05.

Then, the effect of downregulation HULC on osteosarcoma cells was investigated. First, HULC was knockdown in osteosarcoma cell lines successfully (Figures 2(a) and 2(b)). In CCK-8 assay, we found that HULC knockdown significantly reduced cell proliferation in osteosarcoma cell lines (Figures 2(a) and 2(d)). We used Transwell assay to detect cell migration and invasion. HULC knockdown notably inhibited the migration of osteosarcoma cell lines, including SAOS-2 (Figure 3(a)) and MG63 (Figure 3(c)). HULC knockdown markedly inhibited the invasion of osteosarcoma cell lines, including SAOS-2 (Figure 3(b)) and MG63 (Figure 3(d)).

### 3.3. Overexpression of HULC Increased the Proliferation of Osteosarcoma Cells

**Figure 4:**
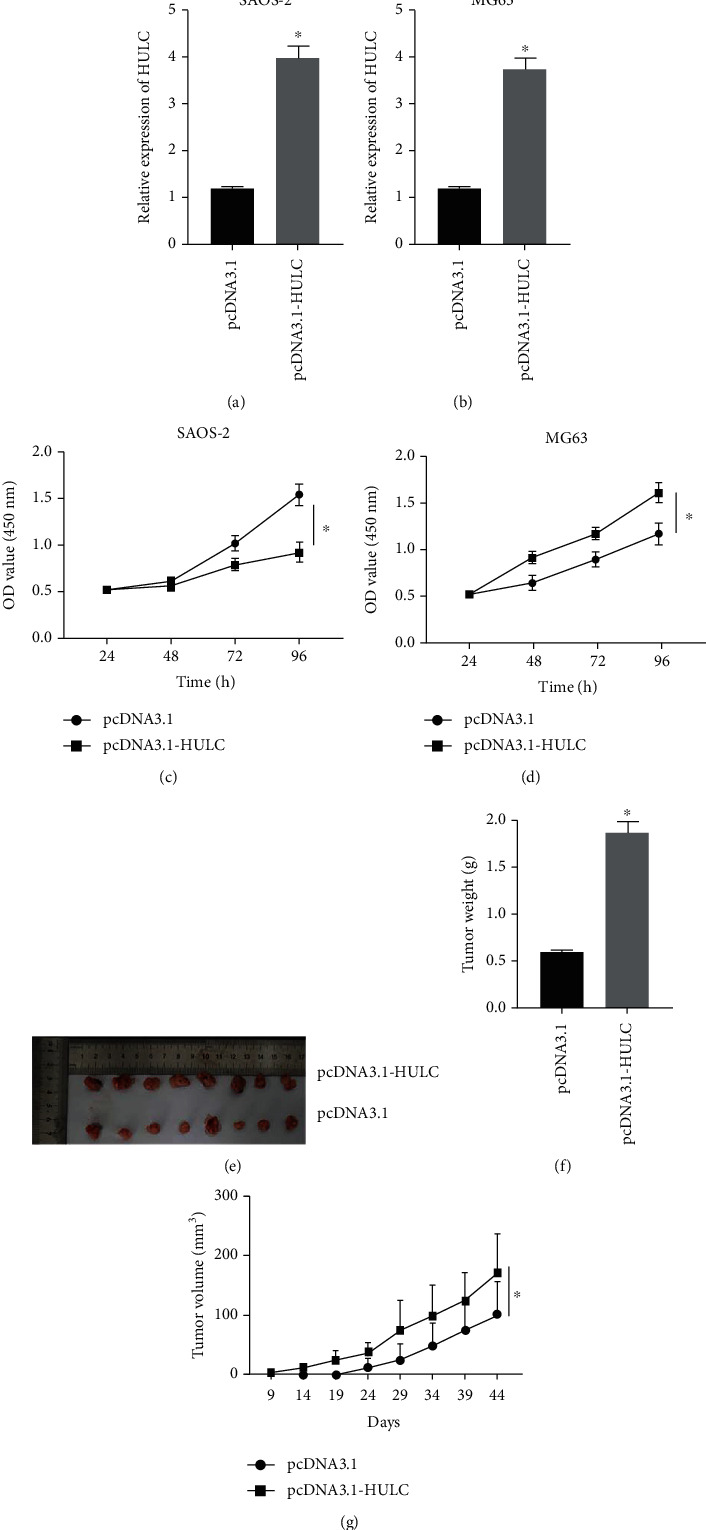
Overexpression of HULC increased the proliferation of osteosarcoma cells. (a, b) HULC expression in cells was detected by RT-qPCR in SAOS-2 (a) and MG63 cells (b). (c, d) CCK-8 assay was applied to measure the cell proliferation of SAOS-2 (c) and MG63 cells (d). (e) Mouse tumorigenesis assay was conducted. (f, g) The tumor weight (f) and volume (g) were detected. ^∗^*P* < 0.05.

Overexpression experiment was performed to detect the effect of HULC in osteosarcoma cell proliferation. In SAOS-2 (Figure 4(a)) and MG63 cells (Figure 4(b)), we successfully overexpressed HULC using overexpression vectors. Overexpression of HULC significantly increased cell proliferation of SAOS-2 (Figure 4(c)) and MG63 cells (Figure 4(d)). Then, we injected the cultured cells into nude mice for tumorigenesis. As shown in Figure 4(e), HULC overexpression markedly raised tumor size. As observed by statistical data, HULC overexpression markedly raised tumor weight (Figure 4(f)) and volume (Figure 4(g)).

### 3.4. HULC Impeded PTEN to Activate AKT-PI3K-mTOR Pathway in Osteosarcoma Cells

**Figure 5:**
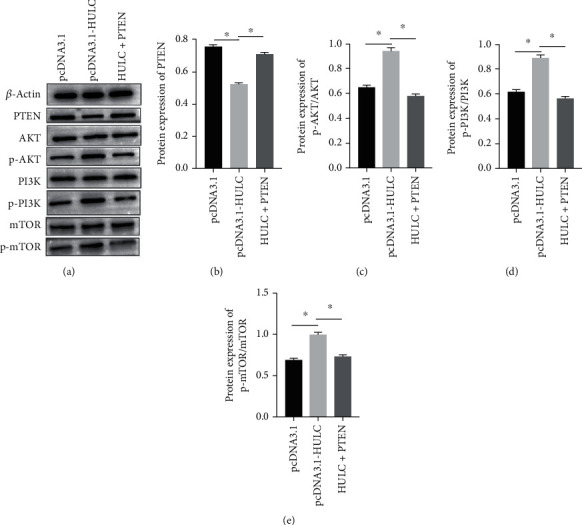
HULC increases activity AKT-PI3K-mTOR pathway by blocking PTEN in osteosarcoma cells. (a) Protein expression was detected by western blot. (b) Overexpression of HULC could result in PTEN downregulation. (c–e) HULC advanced the phosphorylation of AKT (c), PI3K (d), and mTOR (e). ^∗^*P* < 0.05.

In order to investigate the interaction between HULC and PTEN, we overexpressed HULC, and western blot was applied to detect the expression of PTEN and other proteins (Figure 5(a)). We found that overexpression of HULC could result in decreased expression of PTEN protein (Figure 5(b)). Moreover, excessive HULC increased the phosphorylation of AKT (Figure 5(c)), mTOR (Figure 5(d)), and PI3K (Figure 5(e)), while overexpressed PTEN fully abrogated the HULC's action. This suggested that HULC interacts in vivo with signaling pathways that promote cancer formation. However, the overexpression of PTEN cancels the role of HULC. Together, PTEN determined the carcinogenic function of HULC in liver cancer cells.

### 3.5. Overexpressing HULC Enhanced the Malignant Behaviors of Osteosarcoma Cells

**Figure 6:**
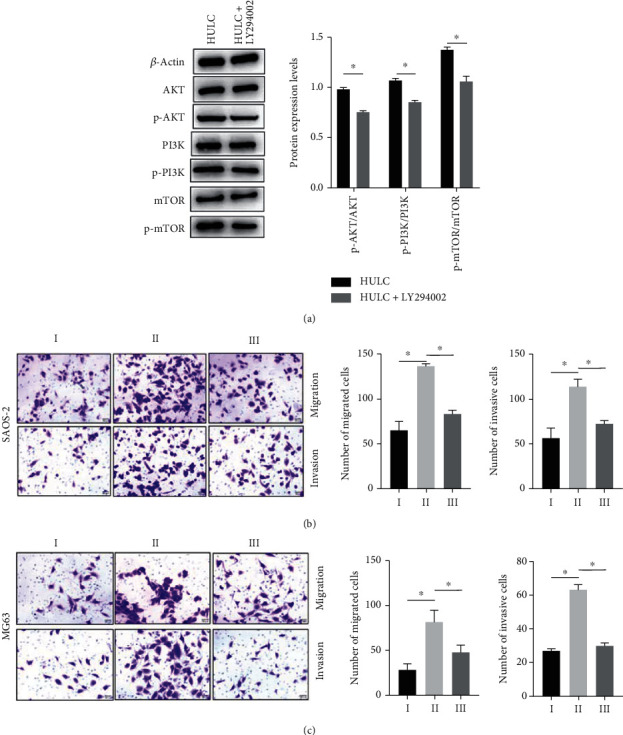
Overexpressing HULC enhanced the malignant behaviors of osteosarcoma cells. I = pcDNA3.1, II = pcDNA3.1 − HULC, and III = pcDNA3.1 − HULC + LY294002. (a, b) Cell migration (a) and invasion (b) assay in SAOS-2 cells was detected by Transwell assay. (c, d) Cell migration (c) and invasion (d) assays in MG63 cells were measured by Transwell assay. ^∗^*P* < 0.05.

To explore whether the effect of HULC on osteosarcoma cells depends on PI3K/Akt/mTOR signaling pathway, we added LY294002, an inhibitor of PI3K signaling pathway. LY294002 inhibited the phosphorylation of AKT, mTOR, and PI3K (Figure 6(a)). Overexpressing HULC enhanced cell migration and invasion of SAOS-2 cells (Figure 6(b)) and MG63 cells (Figure 6(c)), while LY294002 reversed the effects of overexpressing HULC on the cell migration and invasion.

## 4. Discussion

Of all bone cancers, osteosarcoma is the second leading cause of death in children [2]. Osteosarcoma is a fibrogenic malignant bone tumor that can directly or indirectly form tumor bone-like tissue and bone tissue during development, which is a common primary tumor of bone [3]. Osteosarcoma has atypical clinical symptoms in the early stage of onset, which is easy to be confused with other traumatic swelling and pain diseases. Due to the invasive growth of osteosarcoma and early rapid proliferation, the cure rate and prognosis of osteosarcoma are poor. Recent studies have shown that the occurrence, development, and biological characteristics of osteosarcoma are the result of polygenic and multifactorial abnormalities dominated by oncogene activation or tumor suppressor gene inactivation [18]. Therefore, to study the occurrence and development of osteosarcoma, we need to understand the changes of its gene level. For the treatment of osteosarcoma, there are mainly radiotherapy, chemotherapy, and combination therapy, but the prognosis is still not improved [5]. Despite numerous studies, the pathogenic mechanisms of osteosarcoma are still not fully understood.

Previous reports have shown that lncRNA is involved in the formation and development of a variety of tumors [19], mainly because it is involved in important cellular processes such as regulating genome expression, transcription, and translation [20]. lncRNA can participate in the pathological process of tumor cell proliferation, metastasis, and invasion [21, 22]. As the lncRNA located on human chromosome 6p24.3, HULC is primarily located in the cytoplasm and can play an important role in various physiopathological processes by binding to the ribosome. Previous reports have shown that HULC was involved in various processes of tumor formation and metastasis. Plasma HULC is considered to be a biomarker for detecting liver cancer [23]. In gastric cancer, high expression of HULC promotes cell proliferation, inhibits apoptosis of cancer cells, and enhances tumor metastasis [24]. Xu et al. found that HULC regulates PTPRO/NF-*κ*B signaling pathway that promotes the development of lung squamous cell carcinoma [25]. The present study also found that the expression of lncRNA HULC was obviously increased in human osteosarcoma tissues and cells. The present study was investigating the role of HULC in malignant behaviors of osteosarcoma. This study reflected that downregulation of HULC impeded the proliferation, migration, and invasion of osteosarcoma cells. In addition, HULC overexpression markedly increased osteosarcoma cell proliferation and tumor size in vivo. Thus, HULC can promote the malignant behavior of osteosarcoma cells.

PI3 kinase and PTEN are major modulators of the PI3K pathway, regulating cell growth, proliferation, and survival. PTEN regulates PI3K signaling by dephosphorylating the lipid signaling intermediate PIP3 [26]. Mutations in the PTEN gene have been linked to many cancers. Studies have shown that in liver cancer, HULC can inhibit PTEN and accelerate cancer development [15]. Whether a similar mechanism exists in osteosarcoma is unknown. In order to investigate the interaction between HULC and PTEN, we overexpressed HULC, and western blot was applied to detect the expression of PTEN and other proteins. We found that overexpression of HULC obviously decreased expression of PTEN protein. Moreover, excessive HULC increased the phosphorylation of AKT, mTOR, and PI3K while overexpressed PTEN fully abrogated the HULC's action. This suggested that HULC interacts in vivo with signaling pathways that promote cancer formation. However, the overexpression of PTEN cancels the role of HULC. In addition, the present study found that LY294002 inhibited the phosphorylation of AKT, mTOR, and PI3K. Overexpressing HULC enhanced cell migration and invasion of SAOS-2 cells and MG63 cells, while LY294002 reversed the effects. A large number of literature reports pointed out that PI3K/Akt/mTOR signaling pathway is an important signaling pathway in cells, which is widely involved in the regulation of cell functions such as cell proliferation, apoptosis, and invasion [27]. The abnormal activation of PI3K/Akt/mTOR signaling pathway has been confirmed in a variety of cancer cells. Miao et al. believed that PI3K/Akt/mTOR signaling pathway is the key to driving tumor cell proliferation and invasion, and emp1 can promote glioblastoma cell proliferation by activating PI3K/Akt/mTOR signaling pathway [28].

In conclusion, this study supported that the expression of lncRNA HULC was increased in osteosarcoma, which enhanced the progression of osteosarcoma in vivo and in vitro.

## Data Availability

The data that supports the findings of this study is available on reasonable request from the corresponding author.
